# Validity of ultrasound muscle thickness measurements for predicting leg skeletal muscle mass in healthy Japanese middle-aged and older individuals

**DOI:** 10.1186/1880-6805-32-12

**Published:** 2013-09-25

**Authors:** Yohei Takai, Megumi Ohta, Ryota Akagi, Emika Kato, Taku Wakahara, Yasuo Kawakami, Tetsuo Fukunaga, Hiroaki Kanehisa

**Affiliations:** 1 National Institute of Fitness and Sports in Kanoya, 1 Shiromizu, Kanoya, Kagoshima 891-2393, Japan; 2 School of International Liberal studies, Chukyo University, 101 Tokodachi, Kaizu-cho, Toyota-shi, Aichi 470-0393, Japan; 3 College of Systems Engineering and Science, Shibaura Institute of Technology, 3-7-5 Toyosu, Koto-ku, Tokyo 135-8548, Japan; 4 Department of Sports Sciences, Japan Institute of Sports Sciences, 3-15-1 Nishigaoka Kita-ku, Tokyo 115-0056, Japan; 5 Faculty of Health and Sports Science, Doshisha University, 1-3 Tatara Miyakodani, Kyotanabe-shi, Kyoto-fu 610-0394, Japan; 6 Faculty of Sports Science, Waseda university, 2-579-15 Mikajima, Tokorozawa, Saitama 359-1192, Japan

**Keywords:** Dual-energy X-ray absorptiometry, Multiple regression analysis, Bland-Altman plot, Cross-validation

## Abstract

**Background:**

The skeletal muscle mass of the lower limb plays a role in its mobility during daily life. From the perspective of physical resources, leg muscle mass dominantly decreases after the end of the fifth decade. Therefore, an accurate estimate of the muscle mass is important for the middle-aged and older population. The present study aimed to clarify the validity of ultrasound muscle thickness (MT) measurements for predicting leg skeletal muscle mass (SM) in the healthy Japanese middle-aged and older population.

**Findings:**

MTs at four sites of the lower limb and the bone-free lean tissue mass (LTM) of the right leg were determined using brightness-mode ultrasonography and dual-energy X-ray absorptiometry (DXA), respectively, in 44 women and 33 men, 52- to 78-years old. LTM was used as a representative variable of leg skeletal muscle mass. In the model-development group (30 women and 22 men), regression analysis produced an equation with R^2^ and standard error of the estimate (SEE) of 0.958 and 0.3 kg, respectively: LTM (kg) = 0.01464 × (MT_SUM_×L) (cm^2^) - 2.767, where MT_SUM_ is the sum of the product of MTs at four sites, and L is length of segment where MT is determined. The estimated LTM (7.0 ± 1.7 kg) did not significantly differ from the measured LTM (7.0 ± 1.7 kg), without a significant systematic error on a Bland-Altman plot. The application of this equation for the cross-validation group (14 women and 11 men) did not yield a significant difference between the measured (7.2 ± 1.6 kg) or estimated (7.2 ± 1.6 kg) LTM and systematic error.

**Conclusion:**

The developed prediction equation may be useful for estimating the lean tissue mass of the lower extremity for the healthy Japanese middle-aged and older population.

## Background

Age-related loss in leg skeletal muscle mass (SM) accelerates after the end of the fifth decade [1]. This links to the greater influence of aging in the strength capability of the lower compared to the upper limb muscles [2-4], and consequently, to the augmentation of muscular effort in performing the activities of daily living in older individuals [5]. Furthermore, the strength capability of the lower limb muscles is associated with cognitive function [6], and the declines in these abilities lead to disabilities in older people [7]. Therefore, accurate measurement of leg SM in the middle-aged and older population is critical to assess their mobility in daily life.

At present, computerized tomography (CT), magnetic resonance imaging (MRI) and dual-energy X-ray absorptiometry (DXA) are widely used as reference methods for evaluating SM *in vivo*. However, these methods are impractical in field studies examining large populations. Brightness-mode (B-mode) ultrasonography has the same advantage as CT or MRI in visualizing fat and muscle tissues without compression and has been successfully used to evaluate muscle thickness (MT) in an older population [8]. In addition, the B-mode MT measurements are easily applicable in clinical and field surveys with no hazardous effects [9], and the measured values can be significant predictors of limb muscle volume [10,11]. These facts and findings support the applicability of B-mode MT measurements for assessing limb SM in a field survey examining a large sample. To the best of our knowledge, however, the only previous report to have examined the validity of the MT-based prediction equation in a sample including older people is that of Miyatani *et al*. [10]. In addition, their study examined only men and tested only knee extensor muscles. Thus, the applicability of MT measurement for predicting leg SM in older people remains questionable.

The current study aimed to clarify the applicability of ultrasound-based MT measurement for estimating leg bone-free lean tissue mass (LTM) in healthy Japanese middle-aged and older people. To this end, we determined leg LTM by DXA and assumed it to be a valid variable for assessing the leg SM on the basis of previous reports [12-14]. Miyatani *et al*. [11] indicated that ultrasound-based MT measurement was a good predictor of limb muscle volume when combined with limb length. Thus, we hypothesized that the product of MT with limb length should be a strong contributor for predicting the measured LTM.

## Methods

### Subjects

Forty-four women and 33 men, 52- to 78-years old, participated in this study. None of the subjects was or had been an athlete. Moreover, none was using walking sticks or other walking aids and all were functionally independent in daily life. In addition, no participants were on extreme diets or were using any major medications, such as chemotherapy, cardiac, respiratory, or antipsychotic drugs. A hold-out sample validation method was used to develop and test the validity of the new equations. The sample in the current study (n = 77) was split randomly into a model-development group (30 women and 22 men) and a cross-validation group (14 women and 11 men) (Table 1). According to a previous study [15], the ratio of the model-development group to the cross-validation group in the number of the subjects was two to one. This study was approved by the Ethics Committee on Human Research of Waseda University and was consistent with their requirements for human experimentation. The subjects were fully informed of the purpose and risks of the experiment and gave their written informed consent.

**Table 1:** Physical characteristics of the subjects

**Variables**	**Model-development group (n = 52)**		**Cross-validation group (n = 25)**	
	**Men ( n = 22)**	**Women (n = 30)**	**Men (n = 11)**	**Women (n = 14)**
Age, years	62.1 ± 8.6	66.3 ± 5.9	67.5 ± 4.9	63.7 ± 7.7
Height, cm	166.5 ± 5.6	153.5 ± 4.1	166.5 ± 5.6	154.5 ± 3.0
Body mass, kg	65.0 ± 7.3	50.3 ± 5.8	67.0 ± 6.5	53.1 ± 4.9
Body mass index, kg/m^2^	23.4 ± 2.1	21.4 ± 2.5	24.2 ± 2.5	22.2 ± 1.7
Limb length, cm				
Thigh	37.8 ± 1.9	34.8 ± 1.5	37.9 ± 2.0	34.9 ± 1.5
Lower leg	37.0 ± 2.0	34.2 ± 1.7	37.1 ± 2.2	34.1 ± 1.6
MT measurements, cm				
Thigh anterior	4.77 ± 0.53	3.84 ± 0.59	4.56 ± 0.64	3.90 ± 0.57
Thigh posterior	6.12 ± 0.60	4.85 ± 0.48	6.29 ± 0.46	4.98 ± 0.40
Lower leg anterior	3.09 ± 0.24	2.56 ± 0.17	3.05 ± 0.26	2.72 ± 0.28
Lower leg posterior	6.91 ± 0.38	5.75 ± 0.37	6.99 ± 0.61	5.72 ± 0.39
DXA measurements total body				
FM, kg	12.1 ± 3.7	13.8 ± 3.7	13.1 ± 2.5	16.4 ± 2.8
LTM, kg	51.5 ± 4.7	35.7 ± 3.0	52.4 ± 4.9	35.6 ± 2.7
BMC, kg	2.2 ± 0.3	1.5 ± 0.2	2.2 ± 0.3	1.6 ± 0.3
%FM, %	18.2 ± 3.9	26.7 ± 5.0	19.2 ± 2.8	30.4 ± 3.3
Right leg				
FM, kg	1.8 ± 0.6	2.5 ± 0.6	2.0 ± 0.6	3.1 ± 0.7
LTM, kg	8.7 ± 1.2	5.8 ± 0.6	8.7 ± 0.9	6.0 ± 0.6

Values are means ± SDs; thigh length, distance between the lateral condyle of the femur and the greater trochanter; lower leg length, distance between the lateral malleous of the fibula and the lateral condyle of the tibia. BMC, bone mineral content; FM, fat mass; LTM, bone-free lean tissue mass;. MT, muscle thickness; %FM, the percentage of FM in the total mass.

### Muscle thickness measurements

MTs at four sites (thigh anterior and posterior, lower leg anterior and posterior) on the right leg were determined using a real time B-mode ultrasound apparatus (SSD-900, Aloka Co., Tokyo, Japan). The measurement sites were precisely located and marked at the anterior and posterior surface in the middle of the thigh length (the distance from the greater trochanter of the femur to articular cleft between the femur and tibial condyles), and at the anterior and posterior surface in the proximal 30% of the lower leg length (the distance from the articular cleft to lateral malleolus). The length of the segment was determined using a measuring tape. The position of the subjects during the ultrasonographic measurements, the site selected for obtaining cross-sectional images, and determination of MT at each site were the same as those described in previous studies [8,9]. MT at each of the four sites was multiplied by the length of the segment where MT was determined and summed up (MT_SUM_×L).

### Dual-energy X-ray absorptiometry measurements

A whole-body DXA scanner (Delphi A-QDR, Whole body; Hologic, Inc., Bedford, MA, USA) was used to determine fat mass, bone mineral content (BMC) and LTM. The radiation dose was 10 μSv. Lower limb image acquisition and analysis were obtained following the manufacturer’s instructions. Fat mass, BMC and LTM were calculated using software provided by the manufacturer. A radiation technologist performed DXA measurement and data analysis.

### Statistics

In the model-development group, a multiple regression analysis (stepwise) was applied to a set of independent variables which comprised age, sex, and MT_SUM_×L to develop the prediction equation of DXA-based LTM. Sex was coded as a dummy variable: female = 0 and male = 1. The difference between the two variables (measured LTM - estimated LTM) was plotted against the mean values of LTM derived from the two methods to examine for systematic error, as described by Bland and Altman [16]. When the equation was validated, it was applied to the cross-validation group. The standard error of the estimate (SEE) was calculated to evaluate the accuracy of the estimate with the equation. The SEE was expressed as both an absolute value and relative to the mean of the measured LTM (%SEE). Descriptive data are presented as means ± SDs. A Student’s paired t-test was used to test the significance of difference between the measured and estimated LTM. The probability level for statistical significance was set at *P* <0.05. All data analyses were conducted using a statistical software program (SPSS 19.0 for windows, IBM, Japan).

## Results

**Figure 1:**
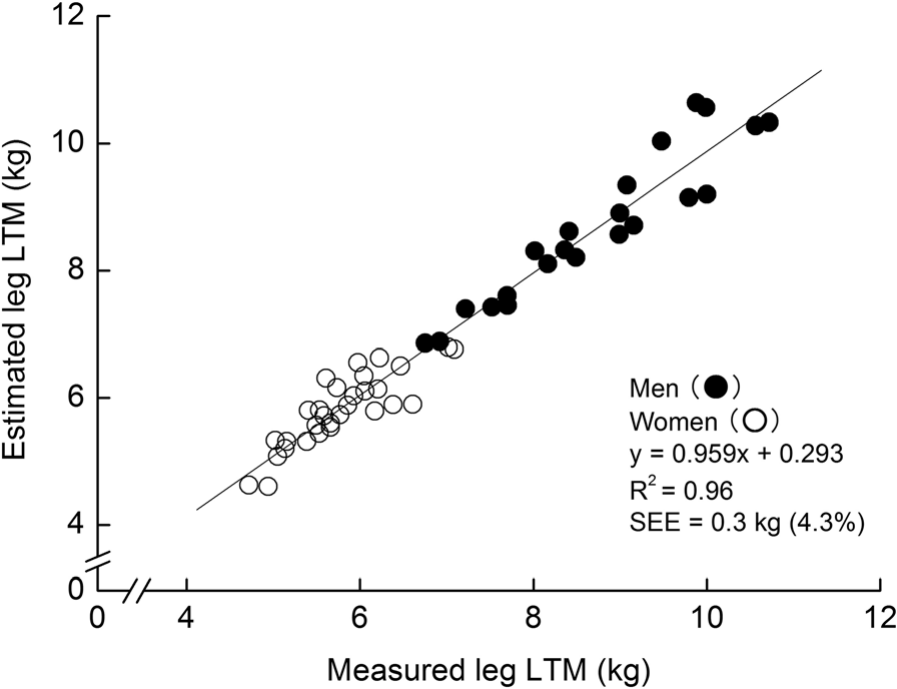
Relationship between the measured and estimated leg lean tissue mass (LTM) in the model-development group.

**Figure 2:**
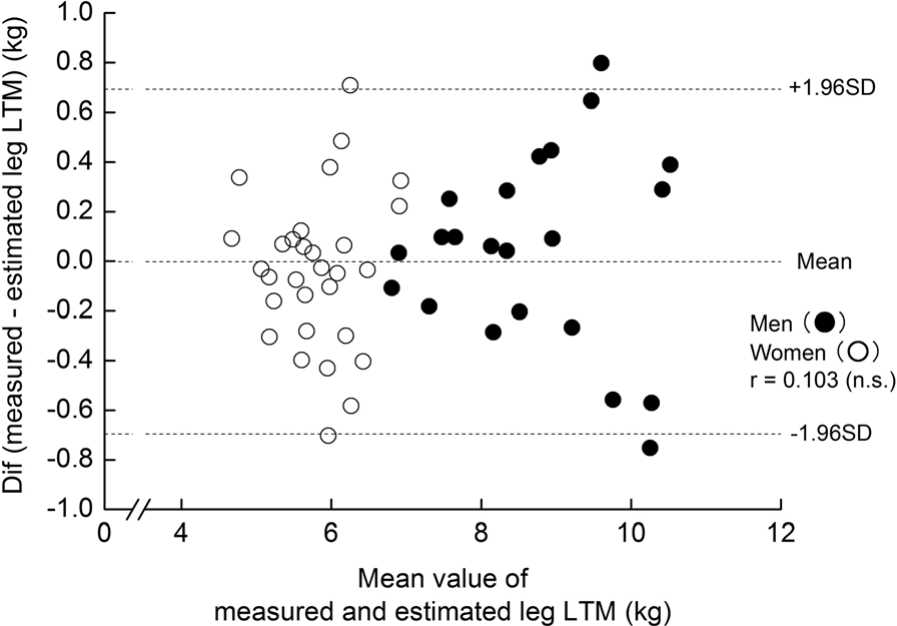
**Bland-Altman analysis for the model-development group.** Difference between the measured and estimated leg lean tissue mass (LTM) (measured LTM - estimated LTM) versus mean values of the measured and estimated LTM. The dotted horizontal lines are mean differences and 95% confidence intervals (CIs).

Descriptive data for the MT and DXA measurements are summarized in Table 1. In the model-development group, multiple regression analysis produced an equation predicting the measured leg LTM, with only MT_SUM_×L as a significant independent variable: leg LTM (kg) = 0.01464 × (MT_SUM_×L) (cm^2^) - 2.767. The R^2^ and SEE of this equation were 0.958 and 0.3 kg (%SEE = 4.3%), respectively (Figure 1). The estimated leg LTM (7.0 ± 1.7 kg) did not significantly differ from the measured leg LTM (7.0 ± 1.7 kg), without a significant systematic error in the Bland-Altman plot (Figure 2). The application of this equation for the cross-validation group did not produce a significant difference between the measured (7.2 ± 1.6 kg) or estimated (7.2 ± 1.6 kg) leg LTM and systematic error (r = -0.012, non significant).

## Discussion

The current results support the hypothesis set at the start of this study, and indicate that ultrasound MT measurements have a high potential for estimating leg SM in healthy Japanese middle-aged and older individuals. This study used DXA-based LTM as a variable representing leg SM. It has been shown that DXA-based appendicular SM models overestimate CT-based SM [17,18]. Despite differences in model formulas, however, all are based on the common principle that most leg LTM is SM [14]. Indeed, leg LTM determined by DXA has been shown to be closely correlated to SM measured by CT [13] or MRI [14]. Furthermore, Kim *et al*. [12] observed a strong association between DXA-based appendicular LTM and MRI-based total SM. These facts and findings indicate that DXA-based LTM is applicable for SM assessment *in vivo*.

In the present study, we used ultrasonography to determine muscle thickness. The ultrasound-based muscle thickness measurements involve not only muscle but also non-contractile tissues such as intermuscular adipose and connective tissues. Intermuscular adipose tissue has been shown to be increased with aging [19]. Thus, there is a possibility that, for older individuals, the muscle thickness measurements might induce overestimation in lean tissue mass.

Besides ultrasonography, prediction equations with the measures of anthropometry [20-22] or bioelectrical impedance analysis (BIA) [23-26] as independent variables have been developed to estimate lower extremity SM. In these equations, the %SEE ranged from 3.3% to 10%. Compared with these values, the %SEE obtained here, 4.3%, is in the lower rank. Among the previous studies cited above, the lowest %SEE values reported for each of the anthropometric and BIA approaches are 3.3% [20] and 3.6% [26], respectively. These values were obtained from prediction equations derived from anthropometric measurements at multiple levels of limbs [20] or multiple BIA along the limb length [26]. With the ultrasound approach, increasing the number of levels for MT measurements will improve the accuracy of LTM estimation.

## Abbreviations

B-mode: Brightness-mode; BIA: Bioelectrical impedance analysis; CT: computerized tomography; DXA: dual-energy X-ray absorptiometry; LTM: bone-free lean tissue mass; MRI: magnetic resonance imaging; MT: muscle thickness; SEE: standard error of estimate; SM: skeletal muscle mass; MTSUM×L: sum of products of MT with the length of segment where MT is determined; %SEE: SEE relative to the mean of the measured LTM.

## Competing interests

The authors declare that they have no competing interests.

## Authors’ contributions

YT, participated in the study design, coordinated research activities, performed statistical analysis, and drafted the manuscript. MO and RA, participated in the study design. EK and TW, measured and analyzed data of muscle thickness. YK and TF, participated in the study design and coordination, and drafted the manuscript. HK, performed statistical analysis, and drafted the manuscript. All authors read and approved the final manuscript.
